# Investigation of optimized observation periods for estimating a representative home range of free-roaming domestic dogs

**DOI:** 10.1038/s41598-023-49851-2

**Published:** 2023-12-20

**Authors:** Filipe Maximiano Sousa, Charlotte Warembourg, Mahamat Fayiz Abakar, Danilo Alvarez, Monica Berger-Gonzalez, Terence Odoch, Ewaldus Wera, Nakul Chitnis, Laura Cunha Silva, Grace Alobo, Maria M. Sikko, Pablo Roquel, Alexis Leonel López Hernández, Salome Dürr

**Affiliations:** 1 Veterinary Public Health Institute, Vetsuisse Faculty, University of Bern, Bern, Switzerland; 2 Graduate School for Cellular and Biomedical Sciences, University of Bern, Bern, Switzerland; 3 Institut de Recherche en Elevage pour le Développement, N’Djaména, Chad; 4 Universidad del Valle de Guatemala, Guatemala City, Guatemala; 5 Swiss Tropical and Public Health Institute, Allschwil, Switzerland; 6 College of Veterinary Medicine, Animal Resources and Biosecurity, Makerere University, Kampala, Uganda; 7 Kupang State Agricultural Polytechnic (Politeknik Pertanian Negeri Kupang), West Timor, Indonesia; 8 University of Basel, Basel, Switzerland; 9 Animal Health Division, Agricultural Department of Sikka Regency, Flores, Indonesia

**Keywords:** Animal behaviour, Epidemiology

## Abstract

Free-roaming domestic dogs (FRDD), as vectors of zoonotic diseases, are of high relevance for public health. Understanding roaming patterns of dogs can help to design disease control programs and disease transmission simulation models. Studies on GPS tracking of dogs report stark differences in recording periods. So far, there is no accepted number of days required to capture a representative home range (HR) of FRDD. The objective of this study was to evaluate changes in HR size and shape over time of FRDD living in Chad, Guatemala, Indonesia and Uganda and identify the period required to capture stable HR values. Dogs were collared with GPS units, leading to a total of 46 datasets with, at least, 19 recorded days. For each animal and recorded day, HR sizes were estimated using the Biased Random Bridge method and percentages of daily change in size and shape calculated and taken as metrics. The analysis revealed that the required number of days differed substantially between individuals, isopleths, and countries, with the extended HR (95% isopleth value) requiring a longer recording period. To reach a stable HR size and shape values for 75% of the dogs, 26 and 21 days, respectively, were sufficient. However, certain dogs required more extended observational periods.

## Introduction

Tracking of animal movements is widely used in several research fields, from animal welfare to conservation biology and public health^1,3,4^. Animal tracking can be a valuable method for gaining insights into the ecology of animals and their interactions with their habitat and other species, allowing a better understanding their behavior. Monitoring movements of free-roaming domestic dogs (FRDD) is of high relevance due to their close contact with humans, other animals and the environment and the potential for transmission of zoonotic diseases^5–8^. Diseases’ transmission simulation models can be improved by better information on behavior, contacts and movements of dogs, contributing to the design of diseases’ prevention and control programs^1,2,9^. Monitoring movements of animals can be done in various ways^3,10,11^, with the usage of Global Position Systems (GPS) devices becoming increasingly relevant over the last years^4,12,13^.

Many studies on dog ecology use the concepts of home range (HR) and utilization distribution (UD)^10,13,14^. The HR can be defined as the area generally used by the individual animal "in its normal activities of food gathering, mating, and caring for young"^15^. The HR size and shape can inform on how dogs move and which areas they visit or use^16–18^. The UD provides information on how areas within the HR are used by the animal. It is calculated based on the relative frequency distribution of locations visited by the animal within the HR area, building a three dimensional kernel^19^. The third dimension represents the density of GPS locations and thus relates to the time the animal spends within a certain area. Using this approach, isopleth lines can be drawn, with the isopleth covering most of the kernel (e.g. 95%) defined as the extended HR and the isopleth covering 50% as the core HR^20^. Each isopleth is characterized by its size and shape and defines areas with similar intensity of presence^21^. By tracking dogs over long enough observation periods, conclusions can be drawn on how the HR changes within different observation period and whether the animal regularly visits new areas or always moves within the same spaces. One question that has been investigated is the possibility of linking certain characteristics of an animal with the size of its’ HR. Several studies tried to provide answers on this topic, but displayed heterogenous findings^14,22–25^. Dogs’ behavioral patterns seem to be complex, influenced by different factors, with distinct individuals displaying heterogeneous behaviors in terms of activity time, areas visited and used and HR size^10,26^. Some dogs display a relatively constant roaming behavior over time, while others display highly variable roaming patterns, with the HR size changing over time^10,11,26–30^*.* At least for some animals, roaming patterns may even show considerable variation between weeks^25^. This is, most probably, influenced by several factors, from extrinsic factors, such as environmental resources or the influence of humans (e.g. whether dogs were raised as shepherd or hunting dogs) to the intrinsic characteristics of the animal itself^10,25,27^.

Studies using GPS devices to track movements of dogs report stark different amounts of recorded time, ranging from hours to weeks^10,13,14,25^. This may lead to biased findings, as excessively short observation periods can lead to the computation of non-representative HR estimates and miss part of the animal’s contacts and places visited. On the other side, too long observation periods will increase the risk of losing GPS devices, leading to a decrease in the sample size and increasing the cost of the study. In this study, data on movements of FRDD from four different countries were collected using GPS devices. The objectives of this study were to calculate the size and shape of the HR from the tracked dogs and to identify a minimum number of recording days required to capture a stable HR value on an individual and population level.

## Materials and methods

### Study sites

This study was conducted within a larger project on dog ecology in four different countries—Chad, Guatemala, Indonesia and Uganda—between January 2018 and March 2019^27,28^ (Supplementary Table S1). Within each country, rural and urban these study sites were selected based on their expected dog densities (low to high) by the local teams^27,28^. Study sites were described in a more detailed way in a previously published study^27^.

### Ethical approval

Ethical approval was requested and granted in each country: in Guatemala by the Universidad del Valle de Guatemala (UVG) International Animal Care and Use Committee (Protocol No. I-2018(3)), the Ethics Review Board of the Committee for Research on Human Subjects of the Center for Health Studies in UVG (Protocol No. 175–04-2018) and by the Community Development Councils of the two rural areas (as it included Maya Q’eqchi’ communities); in Indonesia by the Animal Ethics Commission of the Faculty of Veterinary Medicine, Nusa Cendana University (Protocol KEH/FKH/NPEH/2019/009); and in Uganda by the Uganda National Council for Science and Technology (Protocol NS640). In Chad, the National Chadian Bioethics Committee asserted that no formal ethical approval was needed, according to country's regulation. All the procedures were carried out in accordance with relevant guidelines. Informed consent was obtained from all owners before the start of the study and in accordance with the country-specific regulations.

### GPS units and data collection

In each study site, a 1 km^2^ area was defined using Google Earth. Within the predefined areas, all owned FRDD of more than four months of age were defined as the target population. Pregnant bitches, obviously sick dogs, dogs with necks too large to fit the collar, absent dogs or dogs of an absent owner during all visits, dogs known to be fully restrained and dogs from households where none of the members could read and write (except in Chad) were excluded from the chance of being enrolled. In addition, dogs needed to be large enough to comfortably carry two different GPS collars (the second collar had a short-term (three to five days) GPS device used another study carried in parallel)^27,28^. Following the target population and the exclusion criteria, dogs were selected on a convenient way. In each dog owning household within the study area, the study was presented to an adult member of the family to receive approval for participation. In case this was granted, the dog was collared by a trained team member. In Guatemala only, a monetary incentive of 50 quetzals (6.4 USD) was provided to the participating owners. At the moment of collaring, a table was given to the owners, who were asked to fill with the date, time and place of all human mediated movements of their dogs. Together with the collaring of the dog, a questionnaire to collect information on the dog, dog management and socio-economic status of the household was completed. The interviews were performed in the local language by trained local team members. The data were electronically recorded using the KoboCollect Android application (https://www.kobotoolbox.org/). More detailed information on the questionnaire has been described on a previously published study^28^.

Twenty dogs in Chad, in Guatemala and in Indonesia, and 41 dogs in Uganda were collared with GPS CatLog™ (www.mr-lee.com) units. This type of GPS devices was previously used in studies on FRDD in Australia^20^. The units were kept within a robust plastic cases of around 5 × 5 × 2 cm size and had a total weight of 56 g. Each CatLog unit was identified with a unique number and fixed on nylon dog collars, within a robust plastic case. The place, date and time of collaring was registered for each study dog. To balance battery life with recording periods, the time between two consecutively recorded GPS fixes was set at 15 min. The accuracy of the GPS units was previously assessed, with the mean distance of the recorded locations from their centroid ranging between 14.6 m and 22.8 m (mean of 18.3 m)^10,16^. The owners were asked to remove the collars after, at least, one month (the collar could stay longer on the dog if the owner agreed) and the collars were then collected by a local team member.

### Data management and cleaning

The GPS data recorded and stored in each unit were downloaded using the CatLog Control Center, provided by the producer, and saved as a CSV file. Data were subsequently imported into R (https://cran.r-project.org), which was used for data management, cleaning and analysis. Data from Chad, Guatemala, Indonesia and Uganda were projected from WGS 1984 to local projected reference system UTM 34N, Guatemala Norte, UTM zone 51S and UTM zone 36N, respectively, using R package *proj4*. To reduce the chance of capturing changes in dog's behavior impacted by the collaring or removal of the collar, data from the first and last day of collaring was excluded from the analysis. Datasets with less than 10 recorded days after the removal of the first and last recorded days were excluded from further analysis.

Cleaning of GPS data followed a four-step cleaning process. First, all fixes without latitude and/or longitude values were excluded. The second step was based on the value of horizontal dilution of precision (HDOP), which measures the accuracy of each GPS fix. Higher HDOP values can be translated as less positioning accuracy. Following previous studies we removed fixes with a HDOP value above five^27,31,32^. Third, the speed between each consecutive GPS fixes was calculated and in case speed was higher than 5.7 km/h, the corresponding fixes (the second fix of each pair) were removed. We assumed there would be a maximum limit of speed between fixes and high values would be due to errors. As we could not find a specific plausible speed value for 15 min interval in the literature, the threshold was set based on a plotted histogram of all the calculated speeds, with the exclusion falling on the 0.1% highest speed values. Finally, for each study location, we calculated the degree of the angles formed by each three consecutive GPS fixes. In the representation of movements tracked with GPS devices, acute inner circles are often produced due to error^33^. Therefore, for each study location, we excluded the middle fixes of the 2.5% smallest inner angles. We chose 2.5% as a threshold to minimize the chances of losing correct GPS fixes.

### Data analysis

The process of data analysis and cleaning is presented in Supplementary Fig. S1.

#### Utilization distribution computation

The UD was computed using the biased random bridge (BRB) approach, with R packages *adehabitatHR* and *adehabitatLT*. The BRB approach^34^ was earlier identified as the best suited method for FRDD’s HR calculation using this type of GPS unit^16^. In the BRB function, the parameter *Tmax* was set at five times the capture interval (75 min), the *Lmin* was set as 45 m and the *hmin* was set at 18 m. These values were defined for the CatLog devices in previous a previous study^16^. The UD of each dog was calculated for each day of data collected in a cumulative way, i.e. by using the GPS fixes of that day and of all previous days. For example, the UD of the 7^th^ day included all GPS fixes from day 1 to day 7. From each calculated UD, the size of the 50% (further referred to as core HR), 60%, 70%, 80%, 90% and 95% isopleth (further referred to as extended HR) were retrieved for the further analysis.

#### Home range outcome metrics

To investigate changes in HR size with increasing observation periods, two metrics were calculated for each isopleth and observation period of each animal: a) the HR size, which includes the area within a given isopleth, and b) the shape index, which provides a measure of the HR shape. The shape index was defined as the ratio between the estimated HR size calculated using the BRB method for a given isopleth and the size of the area within the Minimum Convex Polygon (MCP) of that isopleth (Supplementary Fig. S2). The MCP defines the smallest polygon that includes all coordinates (here the limits of the respective isopleth area) and whose angles are all convex^35^. To calculate the MCP for a given isopleth, the coordinates of the external boundaries of the isopleths' polygons were retrieved and the area within the MCP including these boundaries was calculated using the *mcp* function of the *adehabitat* package.

Using this approach, the shape index values ranged between 0 and 1, with values closer to 1 representing more circular and concentrated HR and values closer to 0 more scattered HR. Line plots were drawn to investigate values of these two metrics over increasing observation time periods for each individual dog.

#### Daily percentage of change in HR size and shape

For each metric—HR size and shape index—the percentage of change between the values of each recorded day and its previous was calculated for six isopleths (50%, 60%, 70%, 80%, 90% and 95%) and each individual animal. The percentage of change in HR size for each dog was calculated by dividing the difference between the HR size value at day *i* and the one at day *i*-1 over the HR size value at day *i*-1 (Eq. (1)).

1 $$change \,HR\, size\, value= \frac{{HR\_size}_{i}- {HR\_size}_{i-1}}{{HR\_size}_{i-1}}*100.$$

Similarly, the percentage of change of the shape index was calculated by dividing the difference between the shape index value at day *i* and the one at day *I *− 1 over the shape index value at day *i*-1.

High percentages of change indicate that the additional day of observation brings considerable changes to HR size and/or shape; while small percentages of change over a couple of days indicate that the HR value and/or shape is stabilizing. Line plots were drawn to investigate percentages in change over increasing observation periods for each individual dog.

#### Definition of a minimum required observation period

We hypothesize that for short observation periods, changes in HR size and shape index for each added day of observation will, generally, be large and that with increased observation periods, these changes would decrease, as the HR values for each day stabilizes around similar values. Theoretically, at one point, the percentage of change in HR size and shape index will tend to zero. The HR values could then be defined as representative, as additional days of observation would not substantially change the estimated HR size and shape values. As a complete steady value might be difficult and lengthy to reach – and eventually, even impossible, due to the different factors influencing FRDD moving patterns and the natural changes in behavior over the animal’s life -, especially for higher isopleth levels, we identified, for each animal and isopleth, the number of days after which the percentage of change is equal or inferior to 10% and 20%. We defined this number of days as the minimum required observation period, at a higher (10% change) and lower (20% change) precision. The median, mean and 25 and 75 percentiles of the number of days required to reach the two levels of change (10% and 20%) were summarized amongst all dogs and amongst the dogs per country.

#### Comparisons of the required number of days between countries and sex

To test for the existence of differences in the required number of days for the 10% and 20% maximum change, between countries and sex of dogs, a non-parametric, rank-based statistical test (Kruskal–Wallis for countries and Wilcoxon (rank-sum) test for sex) was applied. Statistical significance was set up at a p value of 0.05.

#### Change in HR size and shape over blocks of days

Finally, we checked for the existence of cases where constant daily changes in the same direction (either increase or decrease) under the daily threshold of 10% or 20%, had a considerable effect on the HR size or shape index values over longer periods of time. We did this by calculating the percentages of change in HR size and shape index values when adding blocks of five and seven days to the previously identified minimum number of days for each dog, for the core and extended HR. As an example, for animal D011 in Guatemala, the number of days after which all daily changes of the core HR size values were equal or below 10% was seven. Hence, for the block of five days, we calculated the percentage of change between the HR sizes at day 7 and day 12, day 12 and day 17, day 17 and day 22 and so on for the entire recorded period. Then, the minimum number of five-days blocks necessary to reach a constant percentage of change equal or under 10% and 20% were identified. The same was done for the change in the shape index value. Animals not reaching a constant value under the thresholds during the daily analysis or without enough remaining recorded days for a day-block comparison (for example, if 33 was the identified day and there was just a total of 35 recorded days) were excluded from this analysis.

## Results

### Collected data

From the 101 collars deployed, 50 (49.5%) were retrieved and 46 (45.5%) had recorded data for 10 or more days (Table 1). Loss of devices was observed in all countries, most prominently in Uganda (26 (63%) of the 41 collared devices). After data cleaning, the number of complete days used for UD calculation ranged from 19 to 85 (median 44).

**Table 1:** Summary of the GPS units collared, retrieved and usable for each study site.

Study site		Deployed	Retrieved (%)	Useable data (%)	Median (range) number of analyzed days
Chad	NDakonon	11	6 (54.5)	5 (45.5)	46 (46–62)
	Sinetaye	9	5 (55.6)	5 (55.6)	56 (19–65)
Guatemala	La Romana	4	4 (100)	3 (75.0)	44 (44–47)
	Sabaneta	6	5 (83.3)	5 (83.3)	36 (20–36)
	Poptún	10	6 (60.9)	4 (40.0)	27 (22–34)
Indonesia	Pogon	4	3 (75.0)	3 (75.0)	26 (23–27)
	Hepang	4	0 (0.0)	0 (0.0)	NA
	Habi	10	7 (70.0)	7 (70.0)	27.5 (27–28)
Uganda	Kamuda	6	0 (0.0)	0 (0.0)	NA
	Soroti	35	15 (42.9)	14 (40.0)	48 (38–85)
Total		101	50 (49.5)	46 (45.5)	

*NA* not applicable.

The number of GPS fixes per dog after data cleaning ranged between 1756 and 6117 (median 4376) in Chad, between 1446 and 4026 (2897) in Guatemala, between 1193 and 3027 (2809) in Indonesia and between 2837 and 7979 (4477) in Uganda. A total of 1486 (3.2%) fixes in Chad, 5049 (13.2%) in Guatemala, 3495 (12.4%) in Indonesia and 1932 (2.9%) in Uganda were removed during the cleaning process.

All 46 dogs enrolled were allowed to roam freely. Fifteen were female (one from Chad, three from Guatemala, five from Indonesia and six from Uganda), 30 were male (nine from Chad, nine from Guatemala, five from Indonesia, seven from Uganda). Information on sex was not recorded for one of the animals from Uganda. Age varied between four and 48 months (median 12) in Indonesia and between three and 248 months (48) in Uganda. No reliable information for age was available for the dogs from Chad and Guatemala. All dogs, except one for which the role played was not recorded, were kept as guardians. Furthermore, 19 (41.3%) were kept as pets or companion, six (13.0%) as shepherds, three (6.5%) as hunters and two (4.3%) as source of meat.

### HR outcome metrics

#### Home range size

**Figure 1:**
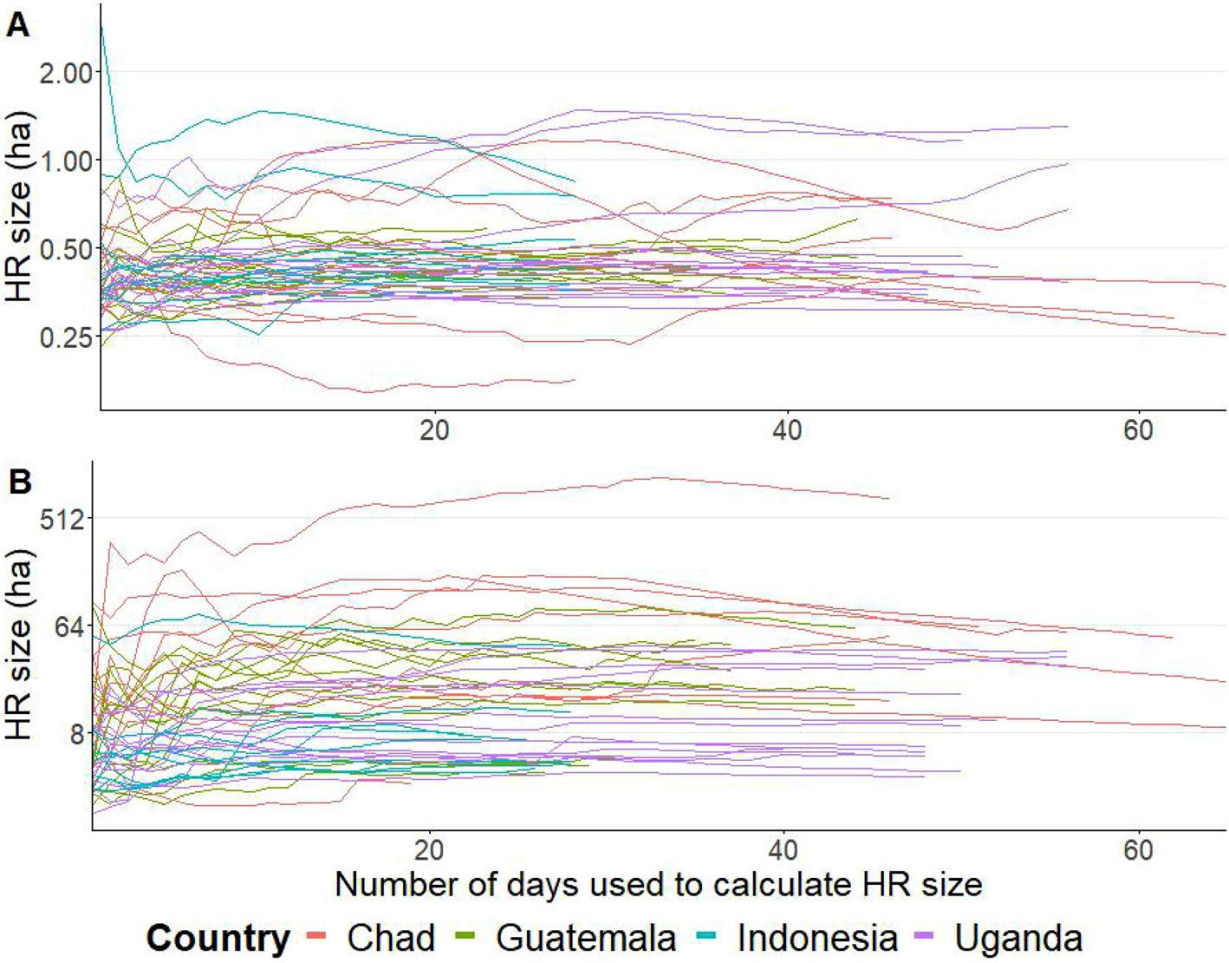
Estimated HR sizes over increasing observation period, for the core HR (**A**) and the extended HR (**B**) for dogs from Chad, Guatemala, Indonesia and Uganda. Each line represents the HR size of one dog and each color represents a country. The Y-axis is presented in a logarithmic scale.

Estimated HR sizes varied along days of observation and between dogs and isopleths (Fig. 1). Generally, for short observation periods, HR sizes changed considerable for each day of additional data considered. The longer the observation period, the higher the number of dogs that seemed to reached a plateau, with little change for each additional day of observation. For the full observation period, which varied from dog to dog, the core HR size ranged between 0.2 and 1.2 hectares (ha) (median 0.4) in Chad (10 dogs), 0.2 and 0.9 ha (0.4 ha) in Guatemala (12 dogs), 0.3 and 2.9 ha (0.4 ha) in Indonesia (10 dogs) and 0.3 and 1.5 ha (0.4 ha) in Uganda (14 dogs). The extended HR size ranged between 2.0 and 1,110.7 ha (50.6 ha) in Chad, 2.0 and 92.7 ha (19.5 ha) in Guatemala, 2.5 and 80.7 ha (5.2 ha) in Indonesia and between 1.7 and 42.9 ha (8.0 ha) in Uganda. The graphs for the 60%, 70%, 80% and 90% isopleths are presented in Supplementary Fig. S3.

#### Shape index

Values of shape index, which were influenced by the spatial dispersion of GPS fixes, also varied with increasing days under observation and between dogs and isopleths (Supplementary Fig. S4). Similarly, to the HR size, the plot lines for each individual dog tended to reach a plateau with increasing observation periods. At the end of the recorded observation period, values of the shape index for the core HR ranged between 0.0 and 1.0 (median 1.0) in Chad, between 0.2 and 1.0 (1.0) in Guatemala, between 0.0 and 1.0 (1.0) in Indonesia and between 0.2 and 1.0 (1.0) in Uganda. The values of the shape index for the extended HR varied between 0.2 and 1.0 (median 0.2) in Chad, between 0.0 and 1.0 (0.2) in Guatemala, between 0.0 and 1.0 (1.0) in Indonesia and between 0.3 and 1.0 (1.0) in Uganda.

### Minimum required observation period

#### Home range size

**Figure 2:**
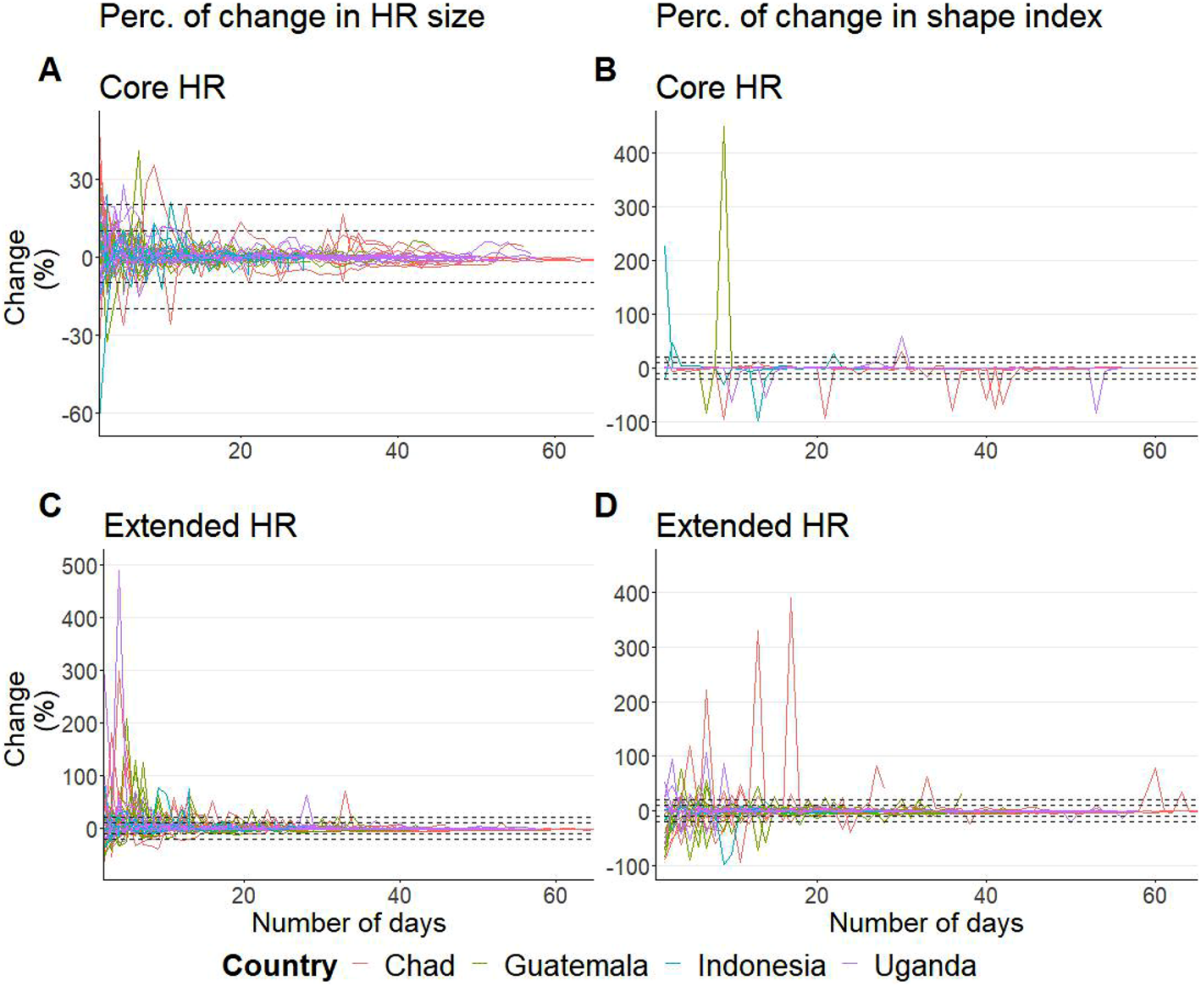
Daily percentage of change in the estimated HR size values for the core (**A**) and extended (**C**) HR and HR shape index values for the core (**B**) and extended (**D**) HR. The dashed lines indicate the 10% and 20% limits of percentage of change. Each line represents the shape index of one dog and each color represents a country. The y-axis is limited to + 500, which excluded some outliers in Fig. C.

**Figure 3:**
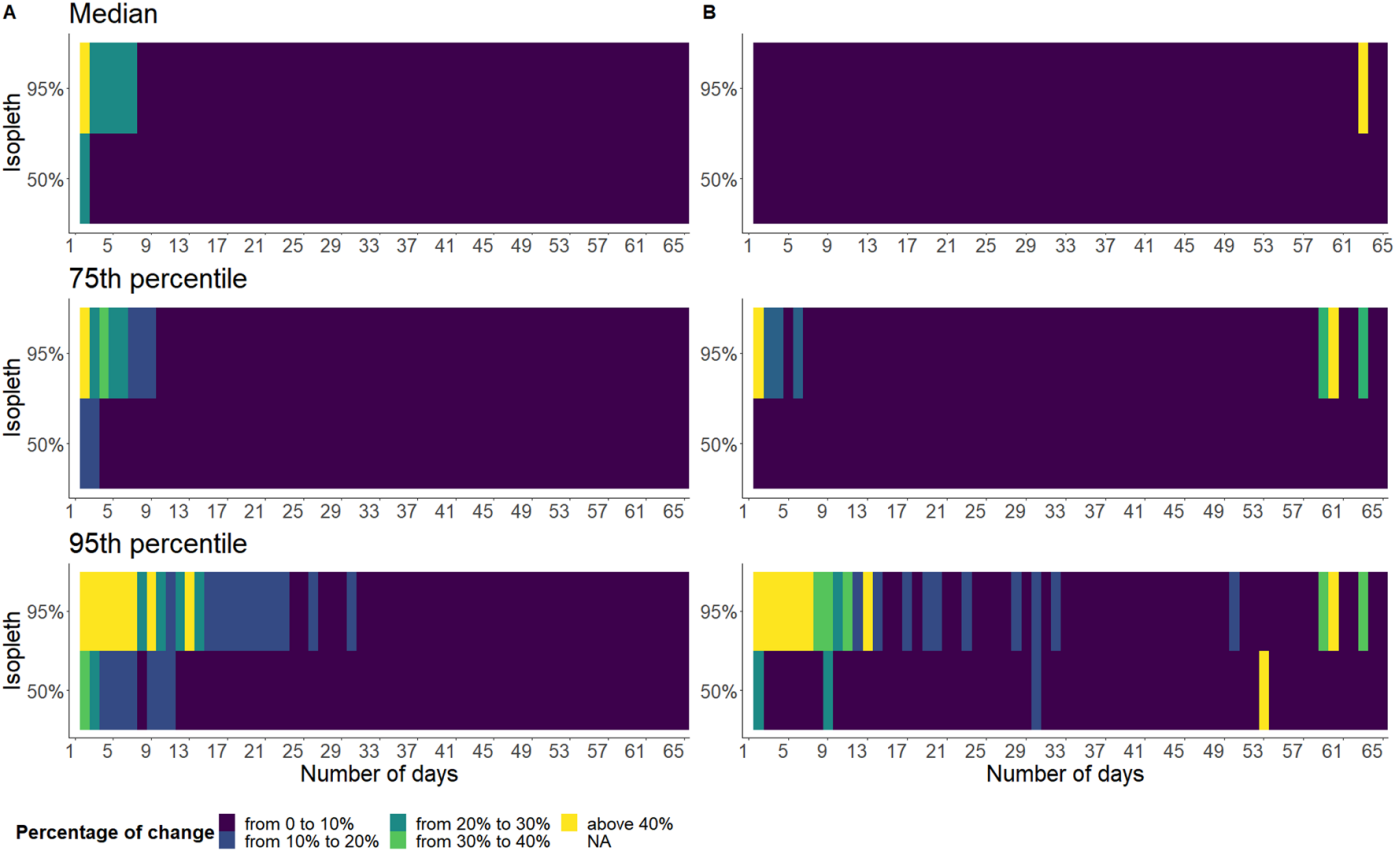
Median, percentile 75 and percentile 95 of the minimum number of days required to reach a stable HR size (**A**) and shape index (**B**) amongst the 46 dogs, depending on the level of percentage of change accepted (from up to 10% to above 40%) and the UD isopleth of interest (y-axis).

The percentage of change in HR size for each additional day of observation was calculated for different isopleths (Fig. 2, Supplementary Fig. S4). Generally, the longer the observation period, the smaller the change for each additional day of observation, with the percentage of change tending towards zero (Figs. 2, 3). The minimum number of days after which percentages of change in HR size for each additional day of observation were of a maximum of 10% and 20% was generally smaller for the core HR than for the extended HR (Fig. 2, Table 2). Some dogs needed to be observed only for 1 day to reach a constant maximum of 10% of change constantly for the core HR, and 2 days for the extended HR (Table 2). However, other dogs needed much larger observation periods, reaching up to 33 and 13 days for the 10% and 20% of change, respectively, for the core HR; and 53 and 34 days, respectively, for the extended HR. For 75% of the dogs, 7 and 2 days were enough to reach a daily maximum of 10% and 20% change for the core HR, whereas 26 and 13 days were required for the extended HR (Fig. 3, Table 2). For the core HR, one dog (2.3%) just reached a constant maximum value of 10% change at the end of the observation period. For the extended HR, one dog (2.3%) never reached a constant maximum change of 10% and four dogs (9.1%) reached it in the last day; one of these four also just reached a constant maximum change under 20% at the last day. The number of days needed to reach a constant percentage of change equal or below 10% and 20% for the other isopleths are presented in Supplementary Table S2.

**Table 2:** Number of observed days required to reach a constant value of percentage of change equal or under 10% and 20% for the HR size and HR shape index for the core and extended HR.

HR	Change (%)	Number of days						Number of dogs without limits reached
		Min	Max	Median	Per. 25	Per. 75	Mean	
HR size								
Core	10	1.0	33.0	3.0	2.0	7.0	5.7	0.0
	20	1.0	13.0	1.0	1.0	2.0	2.5	0.0
Extended	10	2.0	53.0	17.0	10.0	26.0	18.9	1.0
	20	1.0	34.0	7.0	4.0	12.75	9.7	0.0
HR shape index								
Core	10	1.0	53.0	1.0	1.0	7.25	8.5	0.0
	20	1.0	53.0	1.0	1.0	7.25	8.5	0.0
Extended	10	1.0	63.0	6.0	1.0	20.5	13.7	3.0
	20	1.0	63.0	2.5	1.0	13.5	9.9	2.0

Numbers presented indicate the minimum (Min) and maximum (Max), median, percentile 25 (Per. 25), percentile 75 (Per 75) and mean of the number of days amongst all dogs, as well as the number of dogs that did not reach a constant value below the chosen threshold.

#### Home range shape

Similarly to what was observed for the HR size, the longer the observation period, more stable the HR shape index was for most of the dogs (Figs. 2, 3). Therefore, with increasing observation periods, the percentage of change tended towards zero. The minimum number of days after which the percentages of change were constantly up to a maximum of 10% and 20% was smaller for the core HR than for the extended HR (Table 2, Supplementary Fig. S5). There were dogs which only needed to be observed for 1 day for a maximum of 10% of change, for both the core and extended HR (Table 2). However, some dogs needed much larger observation periods, reaching up to 53 days for the core HR and up to 63 for the extended HR, even for the 20% change threshold (Fig. 3, Table 2). For the extended HR, three (6.5%) dogs never reached a constant maximum value of 10% change, and two (4.3%) of those never reached a constant maximum value of 20% change.

### Comparisons of the required number of days between countries and sex

#### Home range size

For differences in the minimum number of days for the HR size between countries, the analysis included 46 dogs (10 from Chad, 12 from Guatemala, 10 from Indonesia and 14 from Uganda) for the 10% and 20% change of the core HR and 20% change in the extended HR; and 45 dogs (10 from Chad, 11 from Guatemala, 10 from Indonesia and 14 from Uganda) for the 10% change of the extended HR (one dog did not reach the defined threshold). No significant differences were found for the core HR size (p = 0.67) and 10% change. However, significant differences were found for the core HR and 20% change (p = 0.02) and for the extended HR size (10% change, p = 0.03; 20% change, p = 0.05).

For the analysis of the influence of the dogs' sex, 15 female dogs and 30 male dogs were included for the core HR and 15 female dogs and 29 male dogs for the extended HR. One dog from Uganda did not have data on sex and therefore was excluded from this analysis. One male dog never reached a number of days after which the percentage of change was constantly equal or under 10% in the extended HR and therefore were excluded from the analysis. The effect of sex was not statistically significant in the core HR (10% change, p = 0.76; 20% change p = 0.61), while in the extended HR was significant for the 10% change (p = 0.012), but not for the 20% change (p = 0.74).

Minimum, maximum, median and mean number of days for each country and UD isopleths are presented in Table 3, Supplementary Figs. S6, S7 and Supplementary Table S2.

**Table 3:** Number of observation days required in each country to reach a constant value of percentage of change equal or below 10% and 20% in HR size for the core and extended HR.

HR	Change (%)	Number of days						Number of dogs with no limits reached
		Min	Max	Median	Percentile 25	Percentile 75	Mean	
Chad								
Core	10.0	1.0	33.0	5.0	2.25	17.75	11.2	0.0
	20.0	1.0	13.0	3.0	2.0	8.75	5.1	0.0
Extended	10.0	15.0	53.0	24.5	17.25	29.75	25.8	0.0
	20.0	2.0	33.0	13.0	10.25	21.25	14.8	0.0
Guatemala								
Core	10.0	1.0	13.0	3.5	1.75	4.75	4.1	0.0
	20.0	1.0	7.0	1.0	1.0	1.25	1.8	0.0
Extended	10.0	6.0	34.0	20.0	14.0	26.0	19.9	1.0
	20.0	2.0	34.0	7.0	6.0	13.5	10.9	0.0
Indonesia								
Core	10.0	1.0	11.0	2.5	1.25	8.0	4.4	0.0
	20.0	1.0	11.0	1.0	1	2.5	2.4	0.0
Extended	10.0	2.0	26.0	11.0	7.25	16.0	11.7	0.0
	20.0	1.0	13.0	3.5	2.0	9.25	5.5	0.0
Uganda								
Core	10.0	1.0	12.0	3.0	2.0	4.0	4.0	0.0
	20.0	1.0	5.0	1.0	1.0	1.75	1.5	0.0
Extended	10.0	4.0	3.,0	21.0	9.25	26.25	18.4	0.0
	20.0	1.0	28.0	6.5	4.0	9.0	7.9	0.0

Numbers presented here indicate the minimum (Min) and maximum (Max), median, mean and percentiles 25 and 75 of the minimum number of days required amongst all dogs as well as the number of dogs that did not reach a constant value below the chosen threshold.

#### Home range shape

For differences in the minimum number of days for the shape index between countries, the analysis included 46 dogs (10 from Chad, 12 from Guatemala, 10 from Indonesia and 14 from Uganda) for the 10% and 20% change of the core HR; 43 dogs (nine from Chad, 10 from Guatemala, 10 from Indonesia and 14 from Uganda) for the 10% change of the extended HR, as three dogs did not reach the defined threshold; and 44 dogs (nine from Chad, 11 from Guatemala, 10 from Indonesia and 14 from Uganda) for the 20% change of the extended HR, as two dogs did not reach the defined threshold. Significant differences were found for both the 10% and 20% change of the core HR (p = 0.02 and p < 0.01, respectively) and for the 10% and 20% change of the extended HR (p = 0.02 and p < 0.01, respectively).

For the analysis of the influence of the sex in the number of days for the shape index, 15 female dogs and 30 male dogs were included for the core HR, 15 female dogs and 27 male dogs for the 10% change and 15 female dogs and 28 male dogs for the 20% change of the extended HR. One dog from Uganda did not have data on sex and therefore was excluded from this analysis. Three male dogs for the 10% change and two male dogs for the 20% change never reached a minimum number of days in the extended HR and were excluded from this analysis. The effect of sex was not statistically significant neither in the core HR (10% change, p = 0.95; 20% change p = 0.95) nor in the extended HR (10% change, p = 0.29; 20% change, p = 0.49).

Minimum, maximum, median and mean number of days for each country are presented in Table 4, Supplementary Figs. S8 and S9.

**Table 4:** Number of observation days required in each country to reach a constant value of percentage of change in the shape index equal or below 10% and 20% for the core and extended HR.

HR	Change (%)	Number of days						Number of dogs with no limits reached
		Min	Max	Median	Per. 25	Per. 75	Mean	
Chad								
Core	10.0	1.0	43.0	28.5	1.0	39.75	22.4	0.0
	20.0	1.0	42.0	28.5	1.0	39.75	22.3	0.0
Extended	10.0	1.0	63.0	23.0	17.0	30.0	25.8	1.0
	20.0	1.0	63.0	17.0	11.0	23.0	20.6	1.0
Guatemala								
Core	10.0	1.0	9.0	1.0	1.0	1.0	1.7	0.0
	20.0	1.0	9.0	1.0	1.0	1.0	1.7	0.0
Extended	10.0	1.0	32.0	14.5	3.0	26.0	15.1	2.0
	20.0	1.0	30.0	13.0	2.0	19.5	12.5	1.0
Indonesia								
Core	10.0	1.0	22.0	1.0	1.0	1.75	4.5	0.0
	20.0	1.0	22.0	1.0	1.0	1.75	4.4	0.0
Extended	10.0	1.0	14.0	1.0	1.0	4.75	3.7	0.0
	20.0	1.0	10.0	1.0	1.0	1.0	2.1	0.0
Uganda								
Core	10.0	1.0	53.0	1.0	1.0	1.0	7.4	0.0
	20.0	1.0	53.0	1.0	1.0	1.0	7.4	0.0
Extended	10.0	1.0	56.0	3.0	1.0	12.0	11.9	0.0
	20.0	1.0	50.0	2.0	1.0	6.5	6.6	0.0

Numbers presented here indicate the minimum (Min), maximum (Max), median, percentile 25 (Per. 25), percentile 75 (Per. 75) and mean of the number of days required amongst all dogs, as well as the number of dogs that did not reach a constant value equal or below the chosen threshold.

### Minimum required observation period considering blocks of days

When calculating the percentage of change in HR size and shape index between blocks of five and seven days, using the previously identified minimum number of days as the starting point, in most cases the percentage of change remained constantly equal or below the thresholds of 10% or 20% (Table 5). A value of zero in the number of days in Table 5 means that the percentage of change remained always equal or below the defined threshold. Yet, in some animals, a constant value equal or below the threshold took longer to be reached or was never reached (Table 5). A value higher than zero indicates the number of extra blocks required to achieve a constant percentage of change equal or below the defined threshold. While for half of the dogs no additional block of days was required (median values is zero for all, except for the 20% change threshold in the five-day block of the extended HR), for 25% of dogs (percentile 75), at least an additional block of five or seven days was needed. Furthermore, in extreme cases, up to nine (10% threshold in the five-day block of the extended HR) blocks were needed in addition.

**Table 5:** Minimum (Min), maximum (Max), median, percentile 25 (Per. 25), percentile 75 (Per. 75) and mean numbers of blocks of five and seven days required in addition to the previously identified number of days to obtain a percentage of change in HR size and shape index equal or below 10% and 20%, for the core and extended HR.

HR	Change (%)	Block (days)	Number of days						Number of dogs included	Number of dogs with no limits reached
			Min	Max	Median	Per. 25	Per. 75	Mean		
HR size										
Core	10	5	0.0	9.0	0.0	0.0	1.8	1.4	45	3
	20	5	0.0	8.0	0.0	0.0	1.0	1.0	45	0
Extended	10	5	0.0	5.0	0.0	0.0	1.0	0.9	36	9
	20	5	0.0	8.0	1.0	0.0	1.5	1.2	42	3
Core	10	7	0.0	6.0	0.0	0.0	1.0	1.1	45	9
	20	7	0.0	5.0	0.0	0.0	1.0	0.7	45	2
Extended	10	7	0.0	4.0	0.0	0.0	1.0	0.6	36	13
	20	7	0.0	3.0	0.0	0.0	1.0	0.7	41	5
HR shape index										
Core	10	5	0.0	1.0	0.0	0.0	0.0	0.0	43	0
	20	5	0.0	0.0	0.0	0.0	0.0	0.0	43	0
Extended	10	5	0.0	4.0	0.0	0.0	0.0	0.5	42	11
	20	5	0.0	3.0	0.0	0.0	0.0	0.1	45	3
Core	10	7	0.0	4.0	0.0	0.0	0.0	0.1	41	1
	20	7	0.0	0.0	0.0	0.0	0.0	0.0	41	0
Extended	10	7	0.0	3.0	0.0	0.0	0.0	0.3	42	11
	20	7	0.0	4.0	0.0	0.0	0.0	0.2	43	5

Dogs without previously identified number of days or without enough remaining recorded days for a day-block comparison were excluded from this analysis. The number of dogs included are presented in column “Number of dogs included” and the number of dogs that did not reach a limit are presented in the column “Number of dogs with no limits reached”.

It is also relevant to notice that some animals never reached a constant value of percentage of change equal or below the defined thresholds within the recording period (Table 5). This is mainly the case for the 10% threshold of the extended HR and seven day-blocks for HR size, with 13 (36.1%) dogs and for the 10% threshold of the extended HR and five and seven day-blocks for the shape index, both with 11 (26.2%) dogs.

## Discussion

In this study, the number of observation days needed to capture a stable HR of 46 FRDD was explored. We calculated the percentage of change of the estimated HR size and shape index values between all observed days, for different UD isopleths and identified the number of days required to reach a constant percentage of change up to 10% and 20%. The number of days depended on the accepted level of percentage of change and isopleth of interest. Especially for lower isopleths (e.g., core HR) stable values for the HR could be achieved relatively quickly, even for the 10% change. For the core HR, 50% and 75% of the dogs reached a stable HR size value within 3 days and 7 days respectively, and a stable HR shape index in within 1 and 7.25, respectively. As expected, it took longer in larger isopleths, with a median of 17 and 6 days for the 10% change in the size and shape, respectively, of the extended HR. Researchers may, therefore, be willing to accept higher changes (and probably lower precision) in HR values to reduce the required observation period. Although, as the day-block analysis additionally showed, it is not easy to determine to all the dogs when a stable HR is reached. From constant small changes in the same direction – increase or decrease – to sudden big changes, some animals will, probably, require an extensive recording interval to capture the complete patterns of activity. While in most cases the duration of the required observation period did not differ considerably from the one-day analysis, up to 36.1% of the animals (in the 10% change threshold for the extended HR) did not reach a constant value equal or below the defined threshold (Table 5).

To our knowledge, this is the first study trying to identify a minimum observation period during which FRDD’s movements should be recorded to capture what could be considered a stable value of the HR. A few studies discussed the need for having a long enough observation period to answer specific research questions^10,23^, but identifying a required number of observation days in a systematic way was still missing. A strength of this study is that it was carried out in four different countries, representing different geographies, environments, role of dogs and people’s attitudes and expectations towards dogs. The HR sizes here estimated are in agreement with the values of HR size estimated in several other studies^16,22,23,25,29,36^.

Our results highlight that the number of days needed to capture a stable HR value varies highly between dogs. This is in agreement with findings from other studies, which separated the studied dogs in different groups based on their HR size and changes over time^10,26^. One study on FRDD in Australia classified dogs in three roaming behavior groups—"stay-at-home", "roamers" and "explorers" –, based on the differences and changes of the HR over time^10^; another, also from Australia, divided the dogs in two groups, "sedentary" and "wandering"^26^. In the current study, such heterogeneity was also observed, even within the same area. Also interestingly, in one previous study, was observed that a dog collared for a longer period of time had an abrupt increase in its HR size after 12 days of relative stability, stabilizing again for 8 days, after which had another increase^25^. This was also observed in our study, as some dogs had sudden increases after reaching constant value of percentage of change during several days. The roaming behavior of FRDD is complex and can be influenced by a combination of a multitude of factors, not only intrinsic to the animal itself, but also probably by the owner's behavior and social, cultural, geographical, seasonal and topographic conditions in the area where the animal lives^10,22,27,28^. Probably this is also reflected in the high differences on the HR sizes found between different animals and countries. Researchers should, therefore, be aware that for FRDD, an observation period of several weeks, or even longer, might be needed to capture a stable HR size for all dogs. However, for the majority (75%) of the dogs, 13 or 26 days, were enough to reach a stable daily value for the 10% and 20% percentage of change of the extended HR, respectively (although the number of dogs considered as having reached a stable value was lower when considering the blocks of days).

Similar results were obtained for the changes in the shape index, a measurement we created of the HR shape, which is impacted by the dog’s visits and time spent in different locations within its’ HR. For some dogs (e.g. in Chad), the number of days required is higher for the core HR than the extended HR (Table 4). This is because the values of the shape index are not only related with the shape of the polygons identified using the BRB method, but also with the dispersion of GPS fixes and the number of polygons for the respective isopleth (Supplementary Fig. S2). In case the animal relocates from one area to other—which might have happened in Chad as some of the dogs were owned by nomad pastoralists –, it might have a bigger impact on the shape index value of the core HR than on the extended HR. This is because the core HR might just include polygons from the main areas of the old and new locations and none in between, changing substantially the shape index value (Eq. (1)). On the contrary, there is a higher probability that the extended HR will include points in between, and therefore more in-between polygons, smoothing this transition. The shape index value of the core HR will, therefore, me more susceptible to changes in location, while the extended HR will be more susceptible to changes in areas visited within the same location. This is reflected on the different spikes observed on Fig. 2, namely around the day 40 for the core HR for the dogs in Chad. This metric may, therefore, be of particular importance for studies focusing on the use of space. The number of days required to reach a stable shape index were similar to those needed to reach a stable HR size, pointing out the relationship between the two metrics.

From the results of the daily and blocks of days analysis, we could confirm that it is not easy to define a specific number of days, or even a narrow interval of days, fitting all dogs.

One of the limitations of this study is the total recording period. Although we tracked the movements of dogs during several weeks, it was still insufficient to estimate the number of days required to reach a stable value for all the dogs. A longer recording period would enable a more in-depth investigation of the HR of dogs with extensive and changing roaming behaviors. Another aspect to consider is that the HR of dogs was investigated in a particular time point in each country. Collaring dogs in different seasons of the year could be helpful in understanding potential seasonal influences on roaming patterns^22,23^. One can also question the feasibility of really identifying the number of days required to capture a representative home range for all dogs. As referred above, besides the natural behavior of the animal, many other factors might influence roaming patterns of FRDD. It might even be that some animals will have relatively long-term irregular changes in their roaming patterns, never really reaching a stable value or requiring quite a long recording period. This can be problematic, as besides the battery capacity limitation, the longer the GPS unit was deployed onto the animal, the higher the risk of loss or damage to the units. This risk was of consequence to our study, with only 50% of the GPS units being retrieved from the collared dogs. This was an issue of particular relevance in Uganda, where the units stayed on the dogs for a longer period compared to other countries. Approaches, such as the one followed by Sparkes et al*.* (2022), where the necessary recording period is calculate based not on the stability of the HR size, but on the maximum value of HR size achieved, could be an alternative. Using this approach, the study, which took place in Australia, suggested tracking dogs for, at least, nine days should be enough. Still, this would not solve the problem of long-term changes for all animals, as some dogs display a stable roaming behavior during some time, followed by sudden changes. Finally, as we sampled dogs based on convenience, only dogs that were accessible and big enough to carry two collars were included. Consequently, our sample might not be representative of the entire population, even if accessible owned FRDD formed the large majority of our study population^27^. Furthermore, we cannot exclude the possibility that the presence of the GPS device caused changes in behavior. It is recommended that the weight of the device does not exceed 5%-10% of the animal’s weight. With the low weight of the device used (56 g), well below this threshold, we do not believe it influenced the roaming patterns of the collared dogs.

In conclusion, this study provides a systematic analysis of the required length of the observation period for FRDD for the estimation of a stable HR values. We suggest that an observation period of two up to 26 days might be enough to capture stable values of HR size for 75% of the dogs; and eight up to 21 days, might be enough to capture stable values of HR shapefor 75% of the dogs. Although, the number of days can be considerably lower, depending on the isopleth level of interest and percentage of change accepted. However, dogs with large variability of roaming patterns were found to require longer observational periods. Further research is needed with longer observation periods to capture the roaming for these dogs. Our findings could be used for guiding future studies on FRDD observation, and it may also be relevant for other free-roaming species kept in close proximity to humans.

### Supplementary Information

Supplementary Information.

## Data Availability

All raw data required to reproduce the results presented in the manuscript are available in the following GitHub repository: https://github.com/Filipe-Miguel/Raw-data-paper and within the article and its supplementary material.
