# Supplementary material for: Investigation of optimized observation periods for estimating a representative home range of free-roaming domestic dogs
Source: Sci Rep. 2023 Dec 20;13:22750. doi: 10.1038/s41598-023-49851-2 (PMC10733426; doi:10.1038/s41598-023-49851-2)
Supplement: Supplementary file 1 — Supplementary Information. [file 41598_2023_49851_MOESM1_ESM.pdf]

“**Investigation of Optimized Observation Periods for Estimating a Representative Home Range of Free-Roaming Domestic Dogs**”

Filipe Maximiano Sousa, Charlotte Warembourg, Mahamat Fayiz Abakar, Danilo Alvarez, Monica Berger-Gonzalez, Terence Odoch, Ewaldus Wera, Nakul Chitnis, Laura Cunha Silva, Grace Alobo, Maria M Sikko, Pablo Roquel, Alexis Leonel López Hernandez and Salome Dürr

Supplementary material

Supplementary Figures

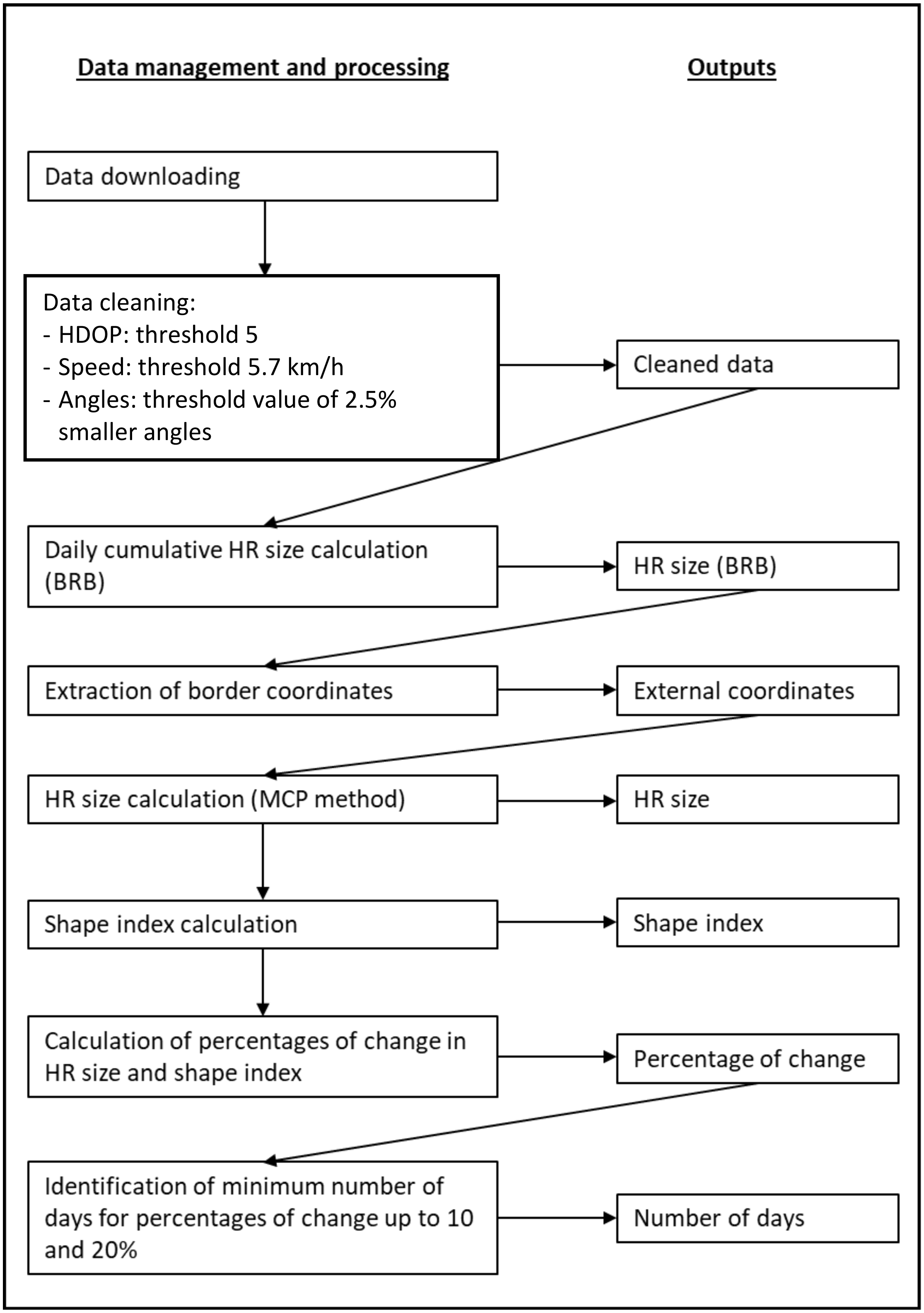

Supplementary Figure S1: Flow chart of data cleaning and management and of the analysis carried on in this study.

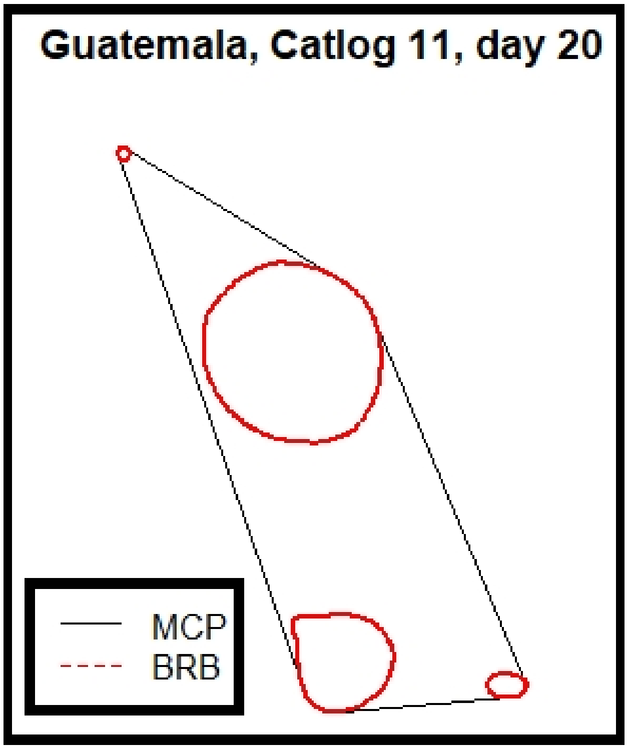

Supplementary Figure S2: Shape of the 95% isopleth for dog D011 from Guatemala, with 20 days of observation. The red lines represent the limits of the 95% BRB isopleth. The isopleth is constituted by four polygons that sum up to an area of 11.7ha. The black line is the MCP that includes the limits of the 95% isopleth and has a size of 35.3 ha. The shape index in this example is 11.7 ha/ 35.3 ha = 0.331.

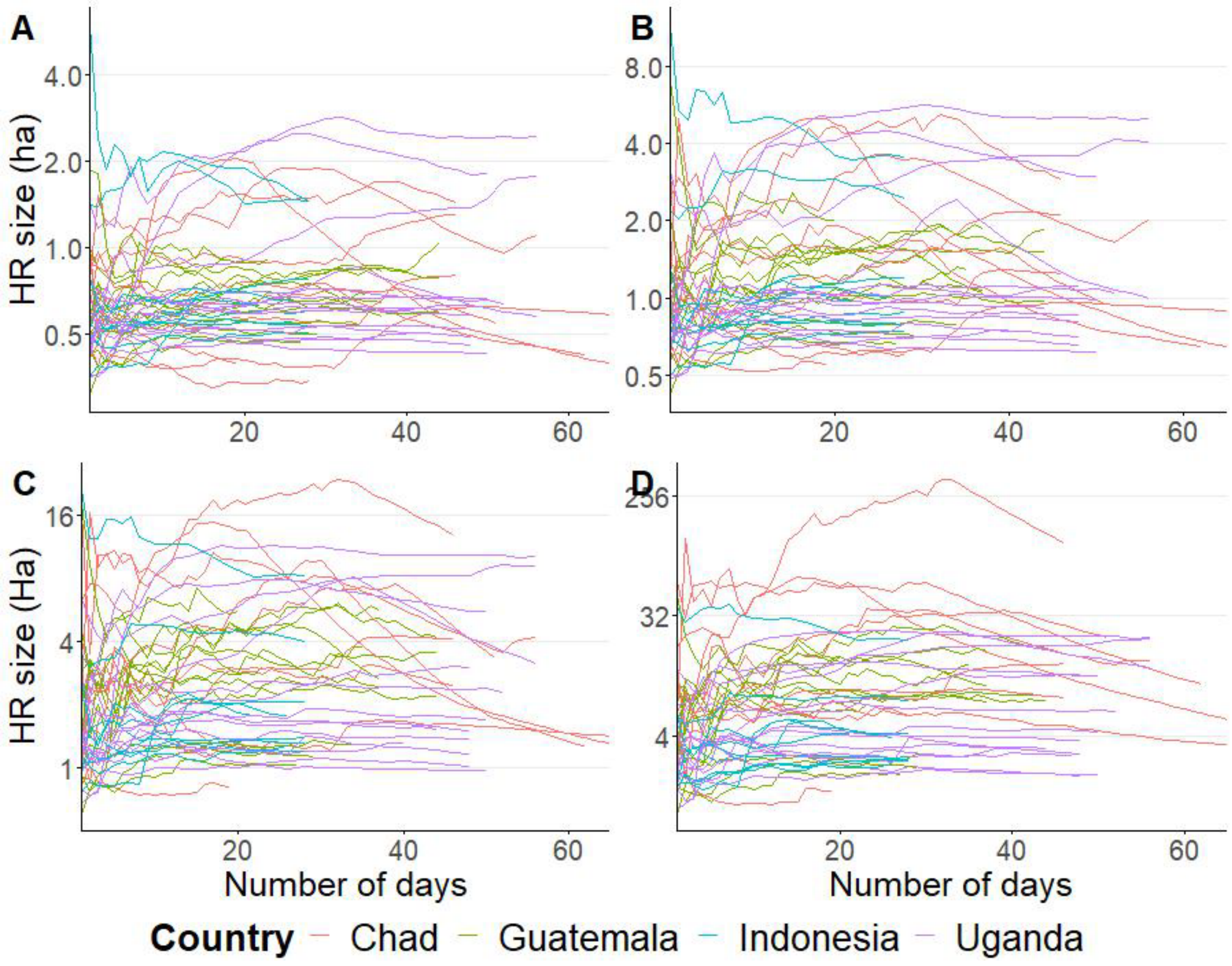

Supplementary Figure S3: Estimated daily HR size values for all the dogs during the study period, for the isopleths 60% (A), 70% (B), 80% (C) and 90% (D) for Chad, Guatemala, Indonesia and Uganda. Y-axis is presented in a logarithmic scale.

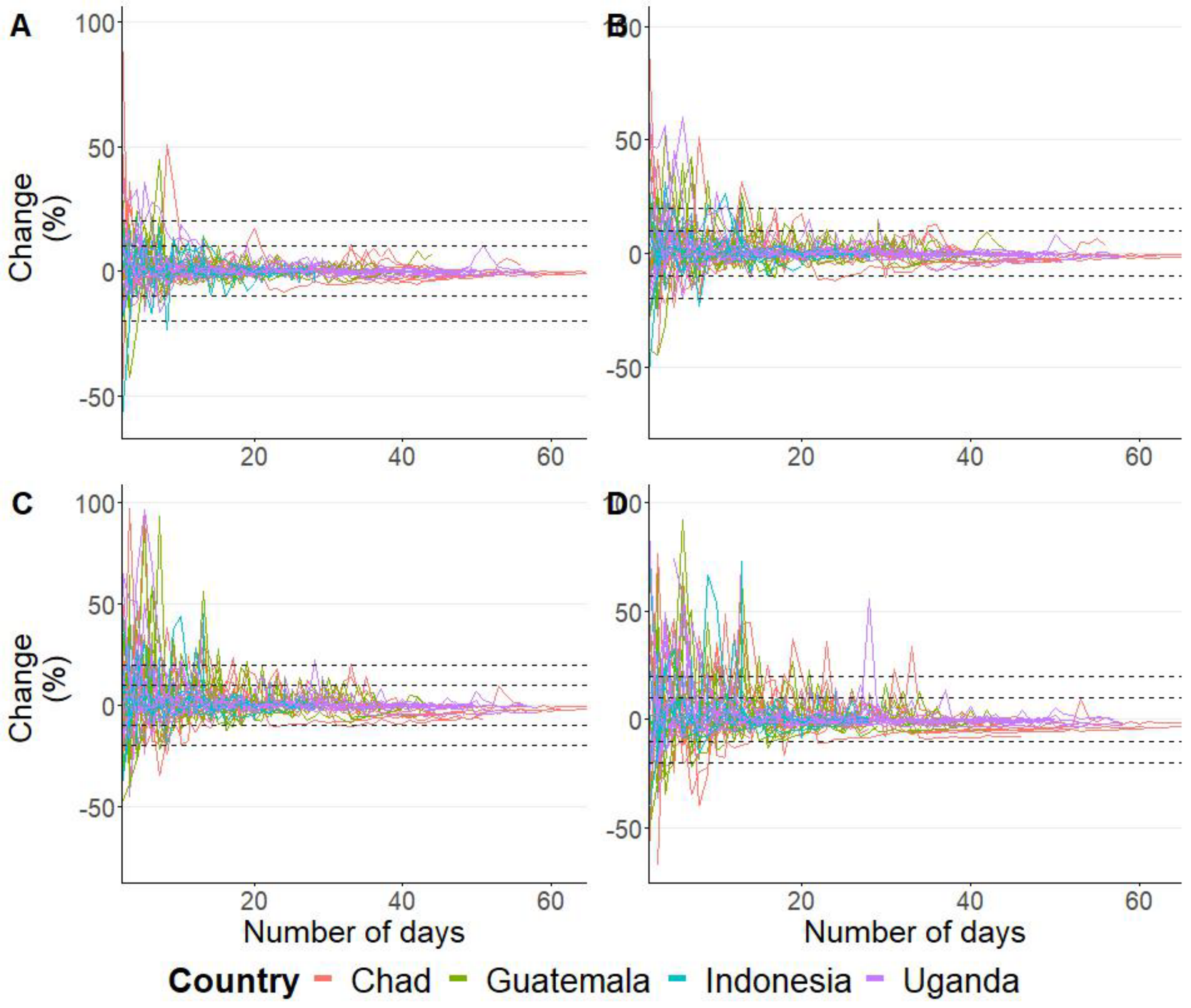

Supplementary Figure S4: Daily percentage of change in the estimated HR size values for the isopleths 60% (A), 70% (B), 80% (C) and 90% (D). The dashed lines indicate the 10% and 20% limits of percentage of change. All dogs from the same country have the same color. The y-axis is limited to +/-100, which excluded some outliers in Figures B, C and D.

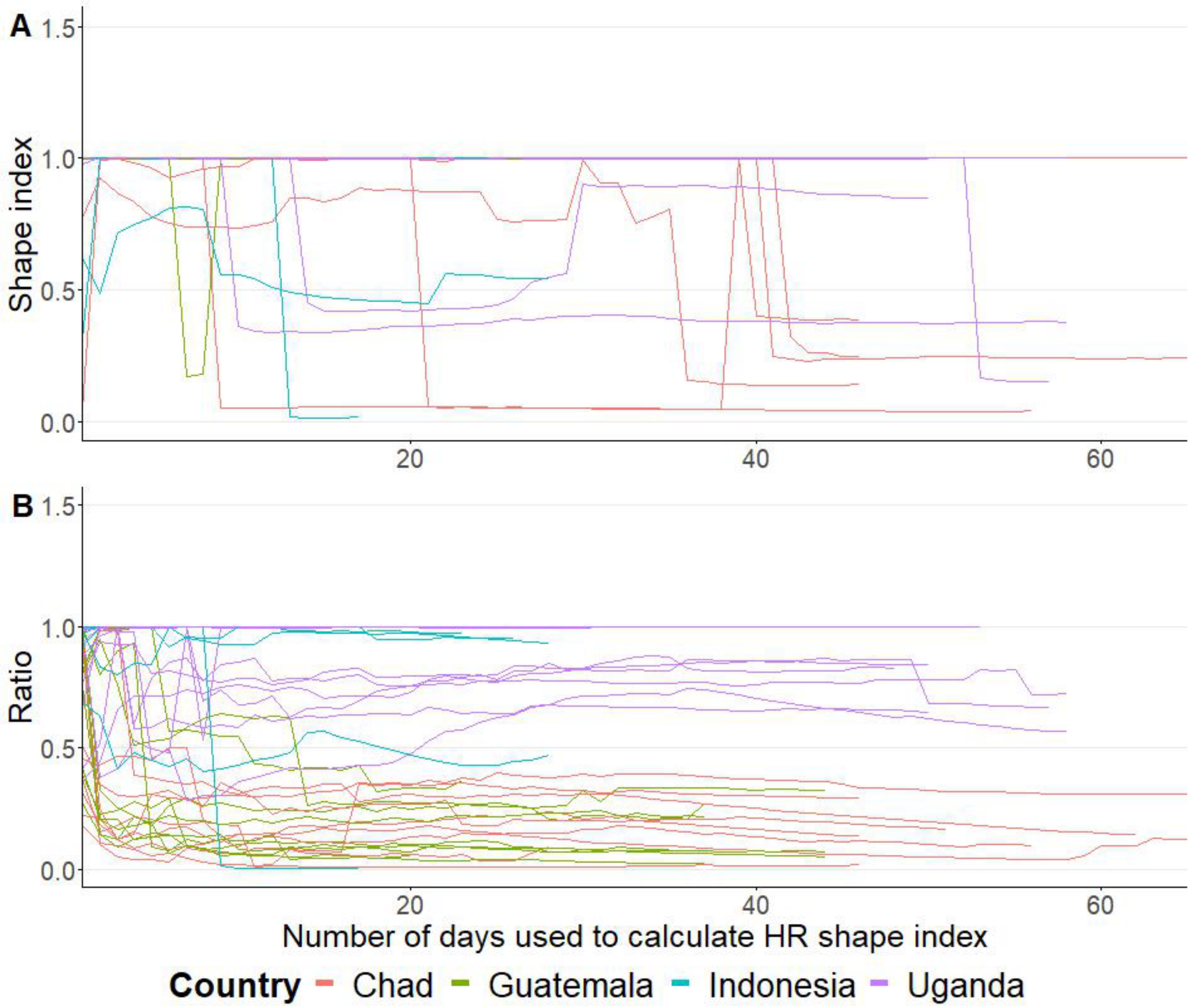

Supplementary Figure S4: HR shape index over the observation period, for the core (A) and the extended HR (B), for dogs from Chad, Guatemala, Indonesia and Uganda. Each line represents the shape index of one dog and each color represents a country.

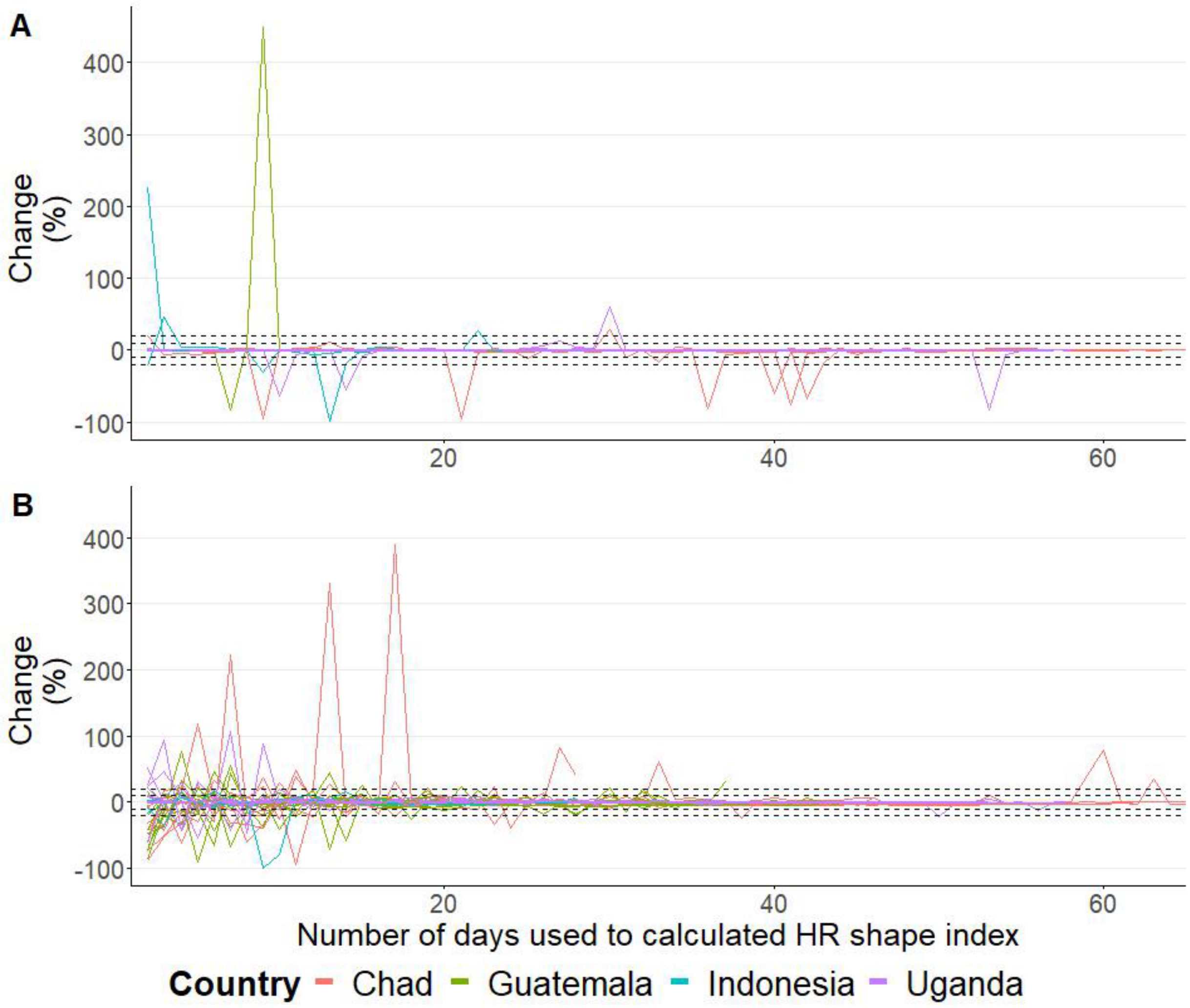

Supplementary Figure S5: Daily percentage of change in shape index values for the core (A) and extended (B) HR. The dashed lines indicate the 10% and 20% thresholds of percentage of change. Each line represents the shape index of one dog and each color represents a country.

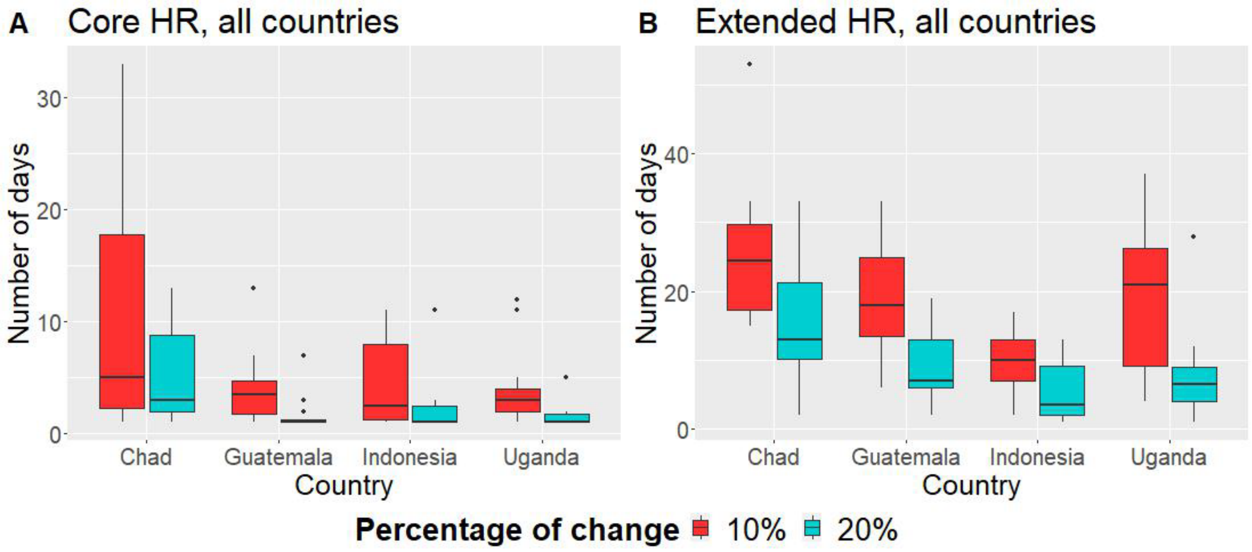

Supplementary Figure S6: Comparison between countries and the number of days required per dog to reach a daily percentage of change in the HR value equal or below 10% (red) and 20% (turquoise) for the core (A) and extended (B) HR sizes. Dogs that never reached the thresholds were excluded.

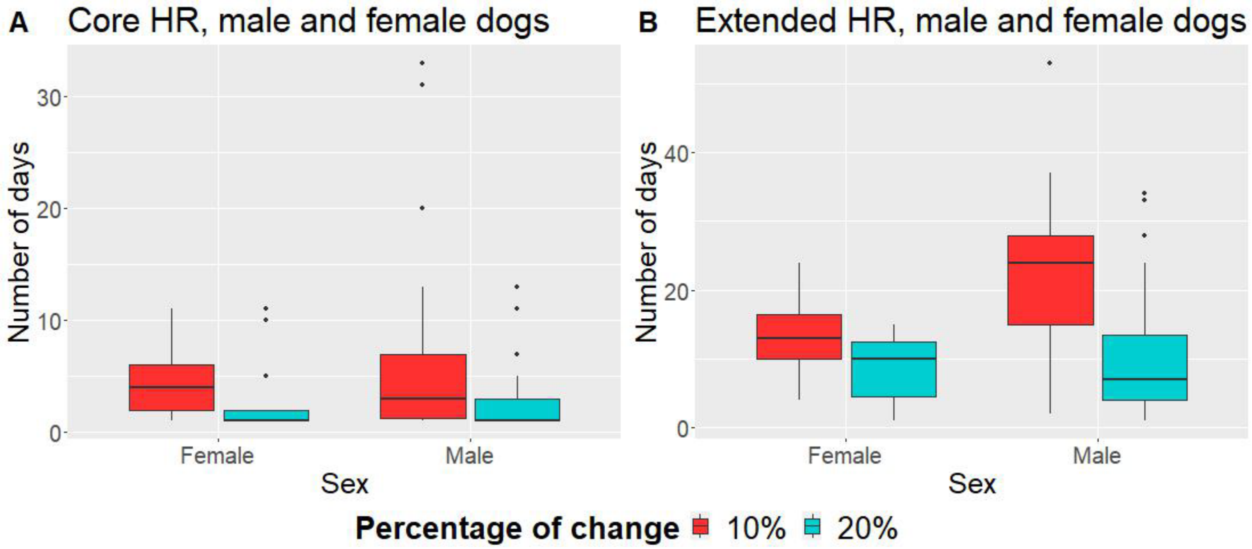

Supplementary Figure S7: Comparison between males and females and the number of days required per dog to reach a daily percentage of change in the HR value equal or below 10% (red) and 20% (turquoise) for the core (A) and extended (B) HR sizes. Dogs that never reached the thresholds were excluded.

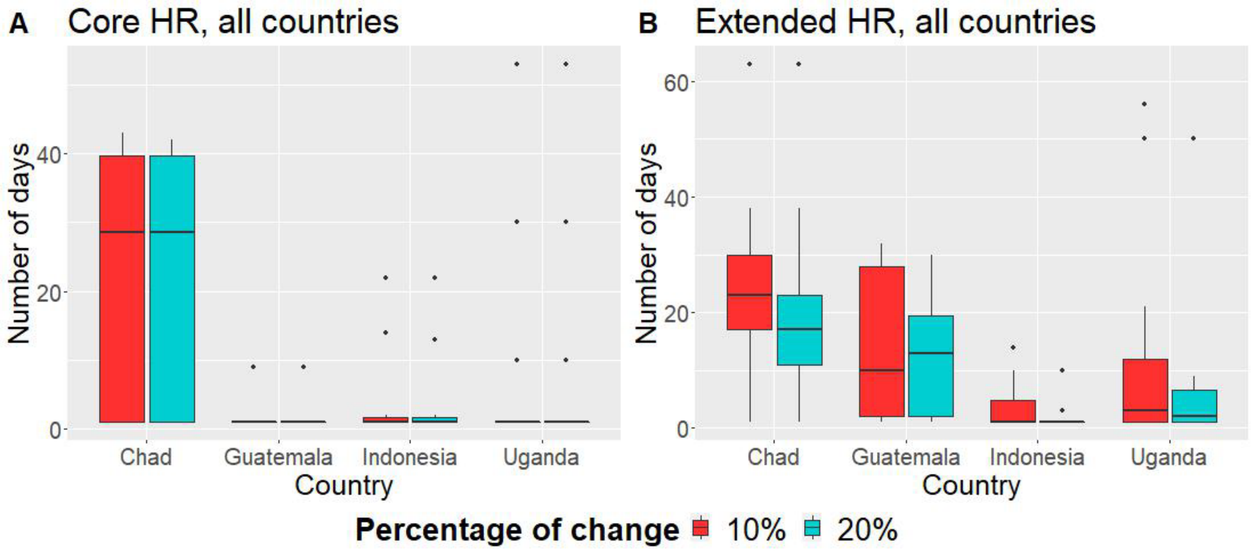

Supplementary Figure S8: Comparison between countries and the number of days required per dog to reach a daily percentage of change in the shape index equal or below 10% (red) and 20% (turquoise) change for the core (A) and extended (B) HR. Dogs that never reached the thresholds were excluded.

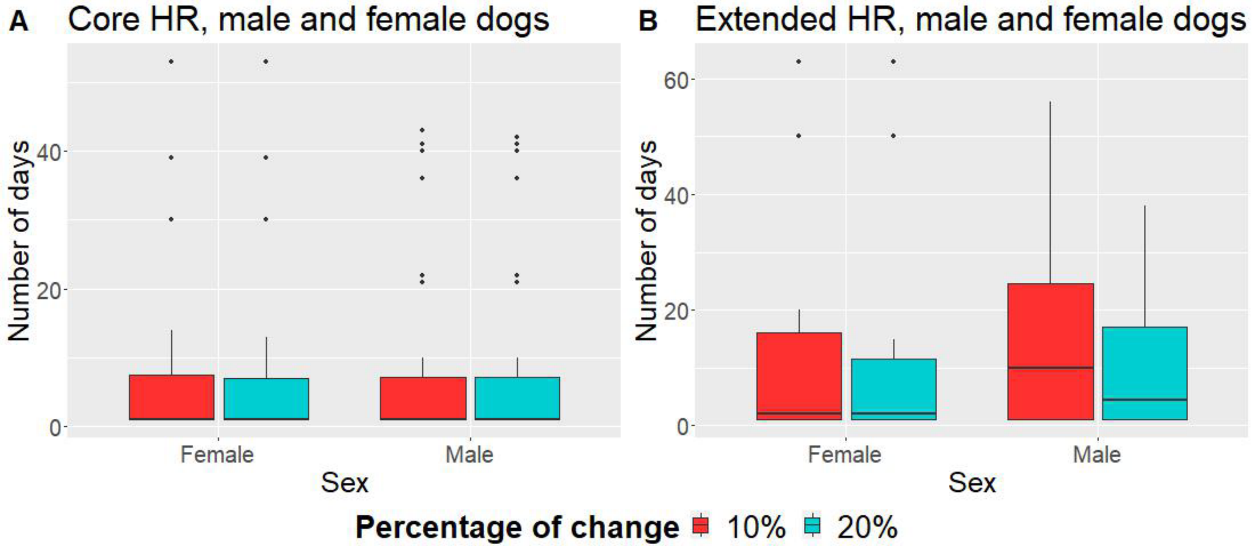

Supplementary Figure S9: Comparison between males and females and the number of days required per dog to reach a daily percentage of change in the shape index equal or below 10% (red) and 20% (turquoise) for the core (A) and extended (B) HR sizes. Dogs that never reached the thresholds were excluded.

Supplementary Tables

Supplementary Table S1: Description of the study sites from where dogs were collared with GPS devices.

Country	Nature of study site	Name of town or village	Expected dogs density	Study period	Coordinates	Admininstrative information
Chad	Rural	NDakonon	Low	Jan-March 2018	8.7475733, 18.53374	Province: Moyen-Chari Department: Grande Sido District: Danamadji
	Rural	Sinetaye	Low	Jan-March 2018	13.2638383 17.721605	Province: Batha Department: Lake Fitri District: Yao
Guatemala	Rural	La Romana	Low	May-June 2018	16.408846, -89.587039	Department: Petén Municipality: Poptún
	Rural	Sabaneta	Medium	May-June 2018	16.324934, -89.324777	
	Urban	Poptún	High	May-June 2018	16.34494, -89.4249067	
Indonesia	Rural	Pogon	Low	July-September 2018	-8.681464, 122.341900	Province: East Nusa Tenggara Island: Flores Regency: Sikka
	Rural	Hepang	Medium	July-September 2018	-8.713159, 122.150506	
	Semi-urban	Habi	High	July-September 2018	-8.6349867, 122.2547617	
Uganda	Rural	Kamuda (I)	Low	Jan-March 2019	1.750024, 33.533461	Region: Eastern Uganda District: Soroti
	Rural	Kamuda (II)	Low	Jan-March 2019	1.703531, 33.556220	
	Semi-urban	Soroti municipality	High	Jan-March 2019	1.7084433, 33.6320767	

Supplementary Table S2: Number of observation days required to reach a constant value of percentage of change in HR size equal or below 10% and 20% for the 60%, 70%, 80% and 90% isopleths. Numbers presented here indicate the minimum (Min) and maximum (Max), median, percentile 25 (Per. 25), percentile 75 (Per. 75) and mean of the minimum number of days required amongst all dogs.

Isopleth	Change (%)	Number of days					
		Min	Max	Median	Per. 25	Per. 75	Mean

60%	10	1.0	51.0	4.5	2.0	9.5	7.7
	20	1.0	9.0	1.0	1.0	3.0	2.4
70%	10	1.0	36.0	7.0	4.0	16.75	11.5
	20	1.0	15.0	2.0	1.0	6.5	4.3
80%	10	1.0	35.0	12.0	6.0	23.0	15.2
	20	1.0	33.0	7.0	2.0	11.75	8.0
90%	10	2.0	37.0	16.0	8.0	24.0	16.9
	20	1.0	33.0	7.5	4.0	12.75	9.5
